# Reply to Letter to the Editor to “Whole-Body MRI, ethical concerns and, equity of access in healthcare: Where we are? Where we are going?”

**DOI:** 10.1093/radadv/umag030

**Published:** 2026-07-14

**Authors:** Preethi Guniganti, Andrea S Kierans

**Affiliations:** Department of Radiology, Weill Cornell Medical College, New York, NY 10021, United States

**Keywords:** WBMRI, screening, early detection, incidental findings

We appreciate the opportunity to respond to the letter “Whole Body MRI, healthcare ethics and access equity: Where we are? Where are we going?”

The authors state that 97% of whole-body MRI (WBMRI) examinations yield incidental findings. It is important to distinguish that only 10% to 25% are clinically significant or require follow-up. These rates are likely to decline as evidence-based guidelines for noncontrast MRI interpretation are widely adopted, several of which we describe, with others under development.

The concern that irrelevant findings medicalize healthy individuals is valid; however, the impact of such findings depends on how we communicate results. At our institution, nurse practitioners deliver results through a patient-centered report that explains findings in accessible language and clearly indicates whether follow-up is necessary. In our experience, when appropriately contextualized, most incidental findings do not cause significant distress or prompt unnecessary follow-up. When findings do require follow-up or intervention, we further reduce patient stress by facilitating appropriate care.

We agree that there is insufficient evidence to support WBMRI as a population-wide screening tool in low-risk individuals, which currently limits payer coverage. However, WBMRI may be cost effective in risk-stratified populations, informed by family history, genetic testing, serum tests, including multicancer early detection assays, environmental or lifestyle factors, and leveraging large datasets with artificial intelligence, an approach we are developing. Ongoing research will further refine which findings warrant intervention versus active surveillance.

Advances in artificial intelligence applied to MRI acquisition and analysis are reducing costs, improving accessibility, and enabling extraction of biomarkers such as body composition and brain volumetrics. This may uncover previously unrecognized links between disease processes and inform prevention strategies, reshaping our understanding of effective screening.

If WBMRI is proven beneficial and cost-effective for selected populations, coverage may expand, as has occurred for conditions such as Li-Fraumeni syndrome. As with many emerging medical technologies, the current self-pay likely reflects an early phase of adoption rather than a fixed model. In the interim, radiologist-led WBMRI screening programs can provide many potential benefits to patients who seek out WBMRI, compared to the predominant corporate model.

